# Cultivating Environmental Ignorance: Non-Circulation of Ethnomedicinal Knowledge about *Culén* (*Otholobium glandulosum*) in the Atlantic World (1646–1810)

**DOI:** 10.3390/plants13202861

**Published:** 2024-10-13

**Authors:** Matteo Sartori, Julia Prakofjewa

**Affiliations:** Department of Environmental Sciences, Informatics and Statistics, Ca’ Foscari University of Venice, Via Torino 155, 30172 Venice, Italy; matteo.sartori@unive.it

**Keywords:** *Otholobium glandulosum*, *culén*, medicinal plants, ethnobotany, ethnomedicine, Chile, Global South, botanical history, plant knowledge, knowledge circulation

## Abstract

A growing body of literature recognises the importance of exploring the uses of plants in historical written sources. The Chilean native plant *culén* (*Otholobium glandulosum*) has a long history of medicinal use, with various parts of the plant, including its leaves, aerial parts, and roots, employed to treat numerous ailments. This study undertakes a Critical Discourse Analysis of historical sources, specifically books published between the 17th and early 19th centuries (1646–1810), focusing on the medicinal applications of *culén*. The research highlights the circulation of plant knowledge over time by adopting an interdisciplinary approach that bridges historical ethnobotany, environmental history, and the history of knowledge and ignorance studies. The study reveals how the evolution of the ethnobotanical and ethnomedicinal knowledge of *culén* reflects the broader environmental ignorance, wherein the scientific community excluded and oppressed the indigenous and local knowledge of the plant. This work underscores the importance of integrating historical studies into modern environmental and botanical research, emphasising the value of understanding past knowledge systems to inform contemporary conservation efforts and sustainable practices.

## 1. Introduction

Studies on the evolution of the relationships between nature and people over the past centuries represent a growing field in ethnobotanical research [1,2]. Recent work by historians and ethnobotanists has particularly emphasised the importance of examining plant knowledge circulation in published sources and the current uses of native and introduced flora fostered by local communities. Investigating the changes in recorded plant uses offers critical insights into the evolving relationship between society and the environment [3,4]. Moreover, historical written sources documenting ethnomedicinal knowledge allow us to explore how records of plant use have transformed over centuries and what factors have influenced these changes [5]. By examining these historical sources, we gain an understanding of how local plant knowledge was interpreted and influenced by broader contexts [6].

In present-day Chile, *yerbateros* (herb sellers) continue selling various plant species along main streets and public squares. Among these, the leaves of *culén*, scientifically known as *Otholobium glandulosum* (L.) J.W. Grimes, a species in the Fabaceae family, is frequently offered [7]. The scientific name *O. glandulosum* was first formally published in 1990 in the *Memoirs of the New York Botanical Garden*. However, in 2023, this name was unplaced from the Plants of the World Online database, meaning its taxonomic placement is no longer recognised within that system [8]. Furthermore, *World Flora Online* currently lists this name as unchecked and is awaiting further taxonomic review [9].

Despite these recent developments, we continue to rely on the *Catalog of the Vascular Plants of Chile* (updated in April 2019) to identify this species [7]. This catalogue remains a widely accepted reference for Chilean flora and provides a stable basis for botanical nomenclature in our work. Nevertheless, this situation highlights the fluid nature of taxonomic classifications, where names and categorisations may shift as new research emerges. However, regional references like the Chilean catalogue often maintain practical authority.

**Figure 1:**
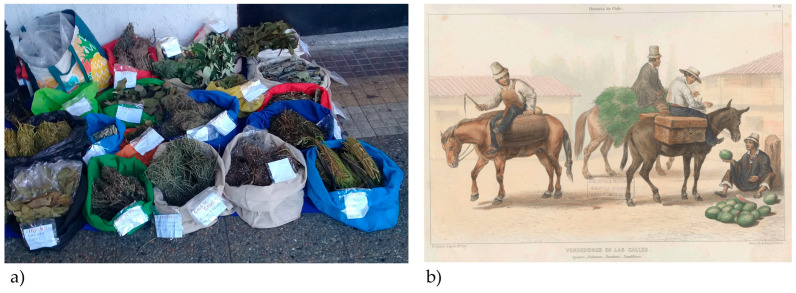
(**a**) Some medicinal plants sold by a herb seller in the Plaza de la Independencia, the main square of Concepción (Chile). Credit by M.S. (2022). (**b**) The French botanist Claudio Gay (1800–1873) engraved in the background of a plant included in his *Atlas de la historia física y política de Chile* (Thunot: Paris, France, 1854, Vol. 1, Plate 48)—a man on horseback, defined as a herb seller, who is carrying some vegetables. Credit: Memoria Chilena (Available online: http://www.memoriachilena.gob.cl/602/w3-article-99629.html, accessed on 25 March 2023).

In Concepción, Chile, herb sellers not only offer the leaves of *O. glandulosum* but also advise customers on their use, continuing a tradition that has persisted for centuries (Figure 1).

Chilean herb sellers often prepare *culén* leaves in tea bags, similar to products offered by a well-known Chilean company run by Mapuche, one of Chile’s indigenous communities. Moreover, the Chilean Ministry of Health has authorised the medicinal use of *O. glandulosum* [10]. Notably, the most frequently cited sources in the Ministry’s catalogue and more recent works on Chilean medicinal plants are the natural histories, travel reports, and descriptions of medicinal plants written during the Chilean colonial period before Chile’s Independence in 1818. Like the herb-seller trade, the ethnomedicinal use of *culén* has endured for centuries. Today, Chilean herb sellers maintain this long-established tradition, fostering a tangible link between society, the Chilean environment, and the evolving practices surrounding *O. glandulosum* [11,12]. The evolution of historical records concerning this plant reveals the dynamic relationship between people and nature, which was shaped by the exchange between Chile and Europe.

The scope of this study is to explore the evolution of the historical, ethnobotanical records of *culén* (*O. glandulosum*) between the 17th and early 19th centuries (1648–1810). The study aims to (1) document the ethnobotanical uses of the plant in historical written sources, (2) trace the evolution of its uses over time, and (3) examine the documentation of local and indigenous practices related to *O. glandulosum* within historical written sources. As recorded in historical sources, the evolution of *culén* ethnomedicinal uses can provide insights into the changes and the influences underlying the ethnobotanical descriptions found in these historical records.

## 2. Results

### 2.1. The Evolution of the Ethnobotanical Uses of Culén

The evolution of *culén*’s uses can generally be divided into two main periods: before and after its spread in Europe by the Franciscan missionaries and later by Jesuits during the 18th century [13]. Previous research has established that the introduction of *culén* to Europe radically changed the recorded uses of this plant [14]. During the colonial period, Chilean indigenous communities used the juice of the leaves of *culén* to heal wounds. However, in the second half of the 18th century, descriptions of the studied species for wound care and general medicinal usage gradually diminished as the plant was introduced in Europe. At the same time, Chilean and European naturalists began recommending *culén* primarily as a recreational tea for European audiences, and the Latin binomial classification began to replace the plant’s common name [14].

Despite these changes, naturalists continued to regard *culén* as a potential remedy throughout the 18th and early 19th centuries. Almost all scientists of the 18th century mentioned the use of *culén* leaves as a treatment for wounds in their writings. For instance, in 1798, the Argentine Jesuit Gaspar Juarez and the Italian abbot Filippo Luigi Gilii highly praised the use of *culén* leaves for healing skin lesions [15,16]. In books published between 1762 and 1810, scholars even recommended the studied plant for treating a broader range of diseases than in the previous period (Table 1 and Table 2).

Furthermore, the use of *culén* leaves in infusions was neither a European invention nor a result of the plant’s spread in Europe. Even before the mid-18th century, Chilean military officer Pedro Pascual de Córdoba y Figueroa and the Spanish Jesuit José Sánchez Lábrador, residing in Chile and Paraguay, respectively, documented the use of *culén* leaves in infusions. Similarly, around 1767, the Spanish Jesuit Miguel de Olivares reported that “*drinking the water from the culén infusion increases the desire to eat*”. In 1774, the English Jesuit Thomas Falkner, who served in Argentina and Paraguay, also noted that the plant possessed the same virtues as tea, particularly as a remedy for indigestion [17].

**Figure 2:**
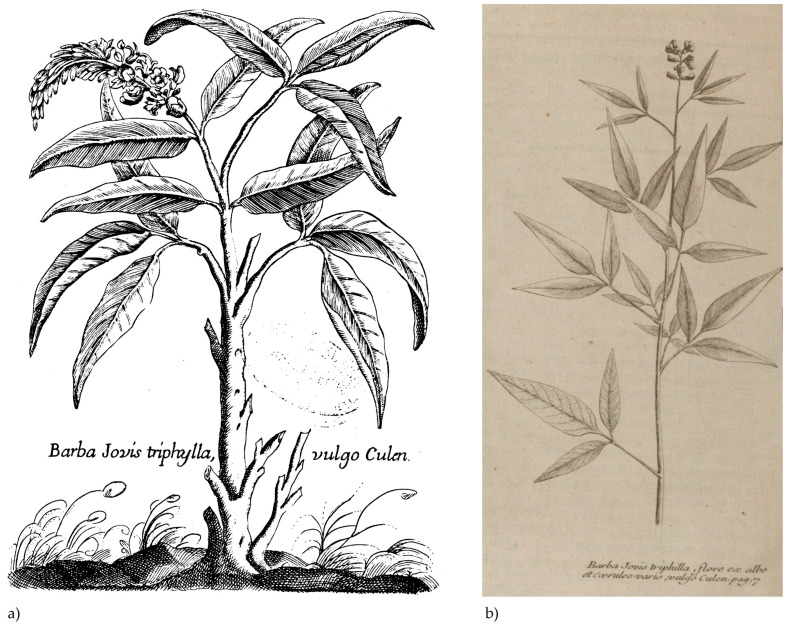
In the engraving (**a**), the Spanish botanist Joseph Quer recorded the *culén* in his *Flora española o historia de las plantas que se crían en España* (Ibarra, Madrid, Spain, 1784, 6, Plate 18) with both scientific and indigenous names. Credit: Biblioteca Digital del Real Jardín Botánico (https://bibdigital.rjb.csic.es). In the picture (**b**), the French botanist Luis Feuillée reported the scientific name he coined “*Barba Jovis triphilla, flore ex albo et caeruleo vario*”, adding the indigenous name “*culen*”.

In 1783, King Carlos III requested that native medicinal plants be sent to Spain. The chief Chilean physician, José Antonio Ríos, organised the expedition and included *culén* among the various species. Ríos recommended the infusion of *culén* leaves and bark to cure stomachache and indigestion. Historical written sources from the same period also documented that the Chilean local communities used the *culén* leaf infusion as a kind of tea for indigestion [18,19]. Thus, the spread of *culén*-based tea was not a result of the plant’s arrival to European territory. However, it was a common practice in Chile even before the introduction of *O. glandulosum* in Europe. Moreover, 18th-century naturalists continued referring to *culén* using its scientific classification and local names [20]. To sum up, naturalists consistently reported the ethnomedicinal uses of *culén* and did not solely identify the studied plant only with its scientific classification (Figure 2).

### 2.2. The Documentation of Local and Indigenous Communities in the Historical Written Sources

During the early colonial period, Spanish chroniclers primarily described the studied plant, reporting its names as “*cuelen*” (likely a misspelling of the actual indigenous name *culén*) and “*albahaquilla*” (meaning “little basil” in Spanish). For instance, Francisco Núñez de Pineda y Bascuñán, a military man from Chillán, mentioned the studied plant in 1629 in his famous work *El Cautiverio feliz*, but without recording any plant use [21].

Generally, no ethnomedicinal information on *culén* (*albahaquilla)* circulated in print until the mid-17th century when Jesuit Alonso de Ovalle published his works in 1646. In his chronicle, Ovalle reported that the leaf juice could be used to heal wounds, citing an instance where a Spanish soldier used it to save his wounded dog. Additionally, Ovalle created a map of Chile for the Spanish King Philip IV, titled *Tabula geographica regni Chile*, in which he depicted a small tree, writing the name “*culén*” and describing it as a “*very healthy plant*” [22].

In the 18th century, French travellers Louis Feuillée and Amédée François Frézier published accounts of their travels in Chile, both of which gained significant attention [23]. Feuillée’s work was translated into German, and Frézier’s book was printed in the Netherlands, each with two editions. The publishing success was probably due to their detailed engravings and the first comprehensive descriptions of the ethnomedicinal uses of Chilean plants. Both French travellers mentioned *culén* in their travel accounts, noting its indigenous use for healing wounds. Frézier even used it to cure himself. Feuillée documented various uses of different parts of the herb by the indigenous communities. However, no authors from that period recorded the possible ethnomedicinal uses of the plant by local communities.

In the second half of the 18th century, scientists continued to report the Latin binomial classifications alongside the indigenous names. However, the local Spanish name “*albahaquilla*” was no longer used to identify the plant. For instance, in the second edition of the *Flora Española* [Spanish Flora], the Spanish physician and botanist Joseph Quer referred to the plant solely as *culén.* Similarly, priests Gilii and Juárez specified that Italians exclusively used the indigenous name. In early historical written sources, scholars documented the indigenous names and practices associated with the plant.

From the second half of the 18th century onwards, most naturalists began including an engraving of the *culén* in their descriptions. This innovation likely facilitated the plant’s knowledge circulation as it spread throughout Europe. However, at the same time, authors increasingly omitted certain aspects of herbal medicinal preparation. The proportion of fragmented uses was higher than in the previous century, limiting the circulation of ethnomedicinal knowledge about the plant.

**Figure 3:**
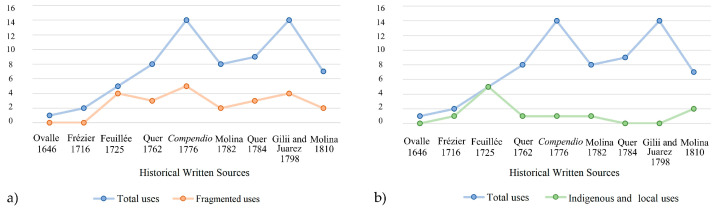
In graph (**a**), the *X*-axis shows the written sources that mentioned the medicinal uses of *culén* (see Supplementary Materials): Ovalle, A. de. *Historica Relatione del Regno di Cile*; Cavallo: Rome, Italy, 1646; Frézier, A.F. *Relation du voyage de la Mer du Sud aux côtes du Chily et du Perou*, *fait pendant les années 1712, 1713 & 1714*; Paris, Jean-Geoffrey Nyon, 1716; 24. Feuillée, L. *Journal des Observations Physiques, Mathematiques et Botaniques*; Giffart: Paris, France, 1725; Quer, J. *Flora española o historia de las plantas que se crían en España*; Ibarra, Madrid, Spain, 1762; *Compendio Della Storia Geografica, Naturale e Civile del Regno del Chile*; Stamperia di S. Tommaso d’Aquino: Bologna, Italy, 1776; Quer, J. Flora española o historia de las plantas que se crían en España; Ibarra, Madrid, Spain, 1784; Gilii, F.L.; Xuarez, G. *Osservazioni fitologiche sopra alcune piante esotiche introdotte in Roma*. Arcangelo Casaletti, Rome, Italy, 1798; Molina, J.I. *Saggio Sulla Storia Naturale del Chili*; Stamperia di S. Tommaso d’Aquino, Bologna, Italy, 1810. The *Y*-axis shows the total number of uses of the studied herb described in each historical text. The blue line represents the total medicinal descriptions, while the orange line corresponds to the partially described knowledge. In diagram (**b**), the *X*-axis represents the written records about the ethnomedicinal knowledge of *culén*. The *Y*-axis shows the total number of uses described in each historical written source. The blue line corresponds to the total medicinal records, while the green line shows the knowledge attributed to indigenous and local communities.

Moreover, Feuillée was the last to explicitly attribute the medicinal uses to the “*natives of the country*”, referring to the indigenous communities [24]. In contrast, the naturalists of the second half of the 18th century did not acknowledge the indigenous origins of these medicinal uses; instead, they treated them as scientific knowledge or inherent properties of the plant. Additionally, many authors failed to report the ethnomedicinal indigenous and local knowledge of *culén* during this time. As a result, after the mid-18th century, indigenous and local knowledge began to disappear from published sources (Figure 3).

Even in the second edition of Molina’s *Natural History of Chile*, the Chilean Jesuit did not fully detail the indigenous and local knowledge of *culén*. He noted that “*Araucanians* [as Mapuche communities were identified at those times] *consider it a panacea, and the Spanish peasants of Chile (…) use it in all their illnesses*” [25]. Despite this, he primarily emphasised the *culén* infusion as a powerful remedy against intestinal parasites, although he acknowledged that other treatments were often preferred for this condition [26].

In 1776, an anonymous work written by a group of Jesuits who had been expelled from Chile stated that “*the Indians (…) apply* [it] *to almost every kind of severe internal illness*” [27]. However, in both cases, the authors failed to specify which parts of the plant were used or for which specific disease the plant was employed. The scientists did not provide detailed instructions on how the indigenous and local communities utilised this remedy.

**Figure 4:**
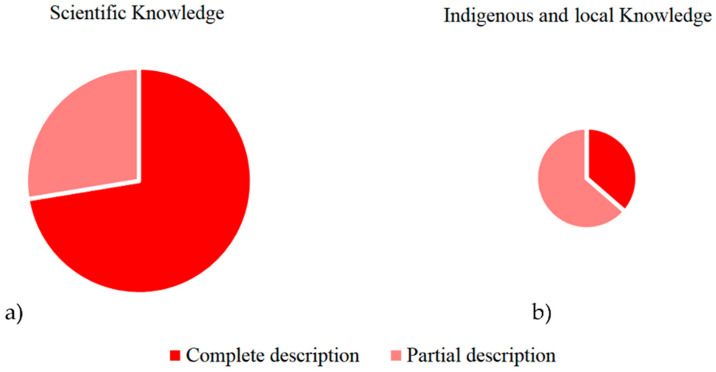
The dark red portions represent the complete records of ethnomedicinal uses of *culén* in naturalists’ books about Chilean flora. The rose parts indicate the incomplete uses reported by the scientists in the historical written sources. The graphics show the fragmentation of the most extensive set of DURs related to scientific knowledge (**a**) and the smallest number of DURs pertaining to indigenous and local knowledge (**b**).

Therefore, like all authors of the studied period, the Jesuits did not fully recognise or give voice to the indigenous and local communities. They primarily emphasised the importance of the native Chilean species that were brought to Italy and the ethnomedicinal uses promoted by the scientific community. Generally, naturalists of the late colonial period prioritised scientific knowledge, documenting more detailed and comprehensive plant uses from a scientific perspective while providing less information on indigenous practices, which were often incomplete or fragmented (Figure 4).

The authors of the 18th century supported the ethnomedicinal knowledge of *culén* but focused solely on scientific descriptions, giving little significance to the uses by indigenous and local communities. They limited the circulation of non-European practices, permanently fragmenting and excluding them. As a result, in the 18th and early 19th centuries, the records of ethnomedicinal knowledge about this plant contributed to the invisibilisation of indigenous and local cultures.

## 3. Discussion

The transformation of knowledge about *culén* into a non-medicinal, tea-like infusion, as mentioned in the introduction, was not the primary transformation that occurred in the public circulation of knowledge about the plant. Whether or not this change happened, and whether it succeeded or failed, the use of *culén* as tea symbolised the exclusion and invisibilisation of non-scientific knowledge. The tea made from its leaves was merely one expression of the coloniality of knowledge, the unbalanced relationship among different forms of knowledge (scientific, indigenous, local, and others) [28].

Overall, the results indicate that the evolution of the knowledge about *culén* is a much more complex phenomenon than just a change in use, a disappearance, or an appropriation of ethnomedicinal knowledge. The recorded practices of *O. glandulosum* were not entirely erased from the historical written sources. For instance, Molina continued to support many uses of that herb for various diseases, even in his last *Natural History of Chile* [25]. The evolution of *culén* ethnomedicinal knowledge was not a straightforward appropriation, as the infusion of the leaves was not directly linked to indigenous knowledge. The most recent use of *culén* leaves as a tea-like infusion may represent a scientific appropriation of local use. Nevertheless, the nearly simultaneous spread of this practice in Chile, Argentina, and Europe makes it difficult to establish clear causal or chronological correlations.

There was a gradual decline in the circulation of indigenous and local knowledge about *culén* throughout the 18th and early 19th centuries. Writers did not promote the non-European ethnomedicinal uses of the plant nor acknowledged the local and indigenous communities in their descriptions. Scientists progressively fragmented the ethnomedicinal knowledge about *culén* and generalised its use, often describing it as a valuable botanical species to cure any disease without explaining how.

One of the most striking findings is that the evolution of the historical ethnomedicinal records of *culén* demonstrates the epistemic exclusion and oppression of non-scientific knowledge. Epistemic oppression refers to the phenomenon that prevents and deprives a person or social group from being able to contribute, directly or indirectly, to the circulation of knowledge [29]. Naturalists of the 18th century did not give equal importance, dignity, or precision enough to the indigenous and local ethnomedicinal knowledge compared to scientific knowledge. This exclusion was not a temporary, isolated phenomenon, limited to a few authors or a scattered group of naturalists. It represented the common denominator of the entire scientific production of the 18th and early 19th centuries. Thus, it was not only a temporary exclusion of local and indigenous knowledge but fundamentally an act of epistemic oppression.

The findings highlight environmental ignorance as one of the consequences of the exclusion and oppression of indigenous and local knowledge in the Atlantic world [30]. A similar impact of coloniality can be observed in the evolution of the ethnomedicinal uses of other Chilean native plants, such as *cachanlagua/cachanlahuen* (*Centaurium cachanlahuen* (Molina) B.L. Rob.) and *canelo/boighe* (*Drimys winteri* J.R. Forst. & G. Forst.) [31]. The ethnomedicinal knowledge of these two plants circulated in published sources during the same period as *culén.* However, they were associated with *Cinnamomum verum* J. Presl and *Centaurium erythraea* Rafn, species familiar to Europeans and linked to related local ethnomedicinal knowledge. The first plant spread as a remedy for fever, while the second circulated as a cure for scurvy. In both cases, the error arose from the perceived similarity between species.

Overall, the circulation of ethnomedicinal knowledge regarding *cachanlagua/cachanlahuen* and *canelo/boighe* was fragmented and eroded, and this rendered the non-scientific uses invisible. According to our study, the Atlantic circulation of public knowledge about *culén* represented a continuous form of cultural oppression. Using *culén* leaves as a recreational tea, rather than as a remedy for wounds, served to exclude and oppress non-European knowledge. This was one of the ways in which indigenous and local communities were erased from scientific works. The overall changes in the ethnomedicinal knowledge of *culén* privileged the Western scientific knowledge, allowing it to prevail over the indigenous and local forms of knowledge.

The non-circulation of local and indigenous ethnomedicinal knowledge contributed to and reinforced a socio-environmental ignorance, which was rooted in a narrow interpretation of diverse knowledge forms. The heterogeneity between scientific and non-scientific medicinal uses of *culén* was not valued. Instead, it was implicitly subordinated and underestimated relative to non-European knowledge and knowers. During the colonial era, Europe became the place where the scientific ethnomedicinal knowledge of *culén* was granted importance and worth. Consequently, the formation of environmental ignorance around the studied species contributed to the formation of the geopolitics of knowledge, where the place of origin primarily and uniquely determined the intrinsic value of knowledge.

During the colonial period, scientists created a deep divide between their knowledge and local and indigenous communities. This divide contributed to the separation, distancing, and marginalisation of other knowledges. Scholars did not include, acknowledge, or integrate these diverse knowledge systems.

Scholars of the 18th century undermined the ethnomedicinal knowledge outside the scientific realm, limiting its diffusion, fragmenting it, and ignoring it. The circulation of public knowledge about Chilean native plants during colonial times was heavily influenced by naturalists’ failure to appreciate any knowledge from outside Europe and any scientific forms as potentially valid and useful.

## 4. Materials and Methods

Plants have long been studied from an environmental history perspective. However, much of the literature has primarily focused on the introduction of species into new territories and the scientific names attributed to them, with an emphasis on flora introduced from Europe to other continents. Additionally, scholars often examine the medicinal properties of plant species without incorporating the knowledge and descriptions provided by local communities or considering the scientific uses of these plants [32,33,34].

The novelty of this study lies in its specific methodology, which centres on ethnomedicinal knowledge reported by various authors and employs an interdisciplinary approach. This approach integrates historical ethnobotany, environmental history, history of knowledge, and history of ignorance, all viewed from a decolonial perspective.

We examined the evolution of historical ethnomedicinal records of *culén* as a phenomenon that reflects societal shifts and represents the changing socio-environmental relationships in the Atlantic world. Our focus was on public knowledge, defined as the information communicated through media and disseminated, utilised, and transmitted via social relations. We explored the close connections between culture and society that develop in public arenas of knowledge, which are specific spaces where certain forms of knowledge are promoted, supported, and fostered. In contrast, others are hindered, fragmented, or rendered invisible [35].

Interpreting the circulation of knowledge as a non-linear process within an epistemic system, we concentrated on public circulation, examining how this knowledge fluctuated over time in various published works, which reported different forms of knowledge [36]. During the Chilean colonial period, multiple forms of knowledge coexisted, primarily indigenous, local, and scientific knowledge. In this context, indigenous knowledge refers to communities that have had historical continuity from pre-European colonisation. Local knowledge pertains to the knowledge of communities directly interacting with their surrounding environments. Scientific knowledge refers to the literature written and cited by naturalists, scientists, travellers, botanists, and doctors.

In alignment with the “ignorance turn”, we considered the coexistence, interaction, and interdependence of knowledge and ignorance. We focused on the fragmentation, erosion, and invisibilisation of knowledge [37]. Central to the decolonial perspective are two main processes that have occurred since the 16th century: the bright side of modernity, characterised by rationality, logic, and the development of scientific knowledge, and the coloniality, the dark side of modernity, in which non-European peoples, knowledges, and ways of being are disregarded by scientists as non-existent [38].

Due to the coloniality of knowledge, the access to, production, distribution, and reproduction of knowledge promoted by scientists and authors were not equal for all knowers. The circulation of knowledge during modern times followed a hierarchical, vertical structure in which specific knowledges were valued more critically than others. This meant that not all knowledge was afforded the same dignity, consideration, or opportunity to spread. During the colonial period, authors promoted this epistemic hierarchy in their writings, favouring some knowledge systems than others. Both the coloniality of knowledge and the epistemic hierarchy, like subterranean rivers, feed the socio-environmental relationship, often showing little visibility and remaining difficult to detect [39].

Recently, some ethnobotanists have shifted their focus toward examining the transformation of plant uses over time and turning to historical sources to highlight how totalitarian dynamics represent complex and articulated phenomena. Simultaneously, there has been a growing focus on environmental and ethnobotanical ignorance dynamics, both in contemporary and colonial contexts [40].

To date, however, research has often framed coloniality solely as a violent form of destruction or as a form of appropriation, sometimes referred to as bioprospecting, of non-European knowledge [41].

This study focuses on the colonial period, beginning from 1646, when Chilean Jesuit Alonso de Ovalle first introduced the knowledge of *Culén* in the Atlantic world, and concluding in 1810, when another Chilean Jesuit, Juan Ignacio Molina, published the second edition of his *Natural History of Chile*. Molina’s book represents the last description of *culén.* Understanding the cultural patterns that influenced the circulation of knowledge about *culén* during the colonial period is crucial to grasping the mechanisms of coloniality of knowledge.

In the colonial descriptions of *culén*, authors did not always include the Latin name of the species, as binomial classification was developed only in the mid-18th century by Carl Linnaeus. However, all colonial authors indicated that they were describing the same herb mentioned by previous writers. Therefore, we considered all naturalists who described the same plant species.

For this research, we employed Critical Discourse Analysis (CDA) to explore the ethnomedicinal discourse surrounding *culén* (*O. glandulosum*) in colonial texts. Data were collected from available colonial sources (see Supplementary Materials) in their original languages, all of which included ethnomedicinal descriptions of *culén*. Nine sources, published between 1520 and 1818, were selected, and the information was compiled into a Microsoft Excel database. Each entry in the database represents a detailed report of the uses of *O. glandulosum* reported in the sources (see [42]). Each use was classified according to the different forms of knowledges reported (ethnomedicinal use, property, or mention), the source of information (books, scientific literature, and local and indigenous knowledge), and the form of information (current or historical usage, use/recommendation to use, and for which disease, classified according to the International Classification of Primary Care (ICPC-2) (updated March 2003)).

Through CDA, we assessed the fragmentation of knowledge by examining the extent to which the sources provided sufficient detail to support the medicinal use of the plant species. Knowledge was considered fully described when the authors provided details such as which parts of the plant to use, how to use them, and for which specific disease. In contrast, knowledge was considered partial when essential details for the practical use of *culén* were missing—for example, when it was merely noted as a general medicinal plant or when only its properties were mentioned without further context.

## 5. Conclusions

This study highlights how the historical records of *culén* (*O. glandulosum*) reflect the dynamics of knowledge circulation and suppression during the colonial period. The fragmentation and marginalisation of indigenous and local knowledge by naturalists in favour of European practices reveal the coloniality of knowledge, contributing to the epistemic oppression of non-scientific traditions. The transformation of *culén* into a tea-like infusion for European consumption exemplifies how ethnomedicinal knowledge was appropriated while indigenous uses were disregarded.

Key scientific gaps remain, particularly regarding the full scope of indigenous and local practices associated with *culén* and other Chilean plants. Further research should prioritise recovering indigenous perspectives on *culén* and other native plants through collaboration with local communities and oral histories. It is crucial to explore how colonial knowledge systems shaped the trajectory of plant-based medicinal research, with an emphasis on decolonising this knowledge for modern application. By integrating local knowledge with scientific research, future studies can contribute to a more complete understanding of Chilean native plants and help bridge the historical gap between indigenous and scientific knowledge.

## Figures and Tables

**Table 1:** Disease categories for which *culén* was recommended in historical records before and after its spread in Europe (see Supplementary Materials), according to ICPC-2.

Sources 1646–1732			Sources 1762–1810		
Skin	Bruise/contusion	S16	General	Pain general/multiple sites	A01
Skin	Skin injury, other	S19	General	Bleeding/haemorrhage NOS	A10
			General	Swelling	A08
			Digestive	Dyspepsia/indigestion	D07
			Digestive	Worms/other parasites	D96
			Digestive	Disease digestive system, other	D99
			Musculoskeletal	Rheumatoid/seropositive arthritis	L88
			Neurological	Headache	N01
			Ear	Ear pain/earache	H01
			Skin	Bruise/contusion	S16
			Skin	Skin injury, other	S19
			Psychological	Psychological disorders, other	P99

**Table 2:** The medicinal properties of *Culén* are mentioned in the works before and after the spread of that plant in Europe.

Sources 1646–1732	Sources 1762–1810
Emetic	Balsamic
Purgative	Emetic
Vulnerary	Digestive
	Purgative
	Vulnerary

## Data Availability

Data are contained within the article and supplementary materials.

## Supplementary Materials

The following supporting information can be downloaded at: https://www.mdpi.com/article/10.3390/plants13202861/s1.

## Abbreviations

DUR	Detailed Use Report
ICPC-2	International Classification of Primary Care
CDA	Critical Discourse Analysis
